# Perceptions of Partner Affect Mediate Affect Contagion in Older Couples' Daily Life: An Experience-Sampling Study

**DOI:** 10.1093/geroni/igab046.1093

**Published:** 2021-12-17

**Authors:** Elisa Weber, Gizem Hueluer

**Affiliations:** 1 University of Zurich, University of Zurich, Zurich, Switzerland; 2 University of Bonn (Germany)

## Abstract

Intimate relationship partners dynamically covary in their affective states. One mechanism through which intimate relationship partners experience and shape each other’s affective states is affect contagion, i.e., the spread of affective states from one person to another. The degree to which social-cognitive processes are involved in affect contagion in daily life remains unclear. The majority of older adults live together with a spouse/partner, and intimate relationships are one of the most important social contexts in their daily lives. Expanding on previous research, we focused on contagion of positive and negative affect between older relationship partners, and examined whether processes of affect contagion were mediated by perceptions of partner affect, i.e., how individuals thought their partners felt at previous moments. We used data from an experience sampling study with 152 older heterosexual couples (304 participants; 65+ years old) who reported on their positive and negative affect, perceptions of their partner’s positive and negative affect, and presence or absence of partners 6 times a day for 14 days. Dyadic multilevel mediation models were used to evaluate our hypotheses. We observed strong evidence that processes of positive affect contagion between partners were mediated by perceptions of partner’s affective states. Negative affect contagion was directed from men to women, but not vice versa, and mediated by perceptions of partner’s affective states. Partner presence was unrelated to processes of affect contagion. Our findings help identify underlying mechanisms of affect contagion and support the notion that perceptions of close others’ emotions might shape our own feelings.

